# Time-Variant Linear Discriminant Analysis Improves Hand Gesture and Finger Movement Decoding for Invasive Brain-Computer Interfaces

**DOI:** 10.3389/fnins.2019.00901

**Published:** 2019-09-26

**Authors:** Johannes Gruenwald, Andrei Znobishchev, Christoph Kapeller, Kyousuke Kamada, Josef Scharinger, Christoph Guger

**Affiliations:** ^1^g.tec Medical Engineering GmbH, Schiedlberg, Austria; ^2^Institute of Computational Perception, Johannes Kepler University, Linz, Austria; ^3^Skolkovo Institute of Science and Technology, Moscow, Russia; ^4^Neurosurgery, Megumino Hospital, Eniwa, Japan; ^5^Guger Technologies OG, Graz, Austria

**Keywords:** brain-computer interface, electrocorticography, movement decoding, linear discriminant analysis, spectral whitening

## Abstract

Invasive brain-computer interfaces yield remarkable performance in a multitude of applications. For classification experiments, high-gamma bandpower features and linear discriminant analysis (LDA) are commonly used due to simplicity and robustness. However, LDA is inherently static and not suited to account for transient information that is typically present in high-gamma features. To resolve this issue, we here present an extension of LDA to the time-variant feature space. We call this method *time-variant linear discriminant analysis* (TVLDA). It intrinsically provides a feature reduction stage, which makes external approaches thereto obsolete, such as feature selection techniques or common spatial patterns (CSPs). As well, we propose a time-domain whitening stage which equalizes the pronounced 1/*f*-shape of the typical brain-wave spectrum. We evaluated our proposed architecture based on recordings from 15 epilepsy patients with temporarily implanted subdural grids, who participated in additional research experiments besides clinical treatment. The experiments featured two different motor tasks involving three high-level gestures and individual finger movement. We used log-transformed bandpower features from the high-gamma band (50–300 Hz, excluding power-line harmonics) for classification. On average, whitening improved the classification performance by about 11%. On whitened data, TVLDA outperformed LDA with feature selection by 11.8%, LDA with CSPs by 13.9%, and regularized LDA with vectorized features by 16.4%. At the same time, TVLDA only required one or two internal features to achieve this. TVLDA provides stable results even if very few trials are available. It is easy to implement, fully automatic and deterministic. Due to its low complexity, TVLDA is suited for real-time brain-computer interfaces. Training is done in less than a second. TVLDA performed particularly well in experiments with data from high-density electrode arrays. For example, the three high-level gestures were correctly identified at a rate of 99% over all subjects. Similarly, the decoding accuracy of individual fingers was 96% on average over all subjects. To our knowledge, these mean accuracies are the highest ever reported for three-class and five-class motor-control BCIs.

## 1. Introduction

A brain-computer interface (BCI) establishes a communication pathway from a person's mind to the environment via brain activity alone (Wolpaw et al., 2002; Wolpaw and Wolpaw, 2012). BCIs operate on brain waves that are usually recorded from the electroencephalogram (EEG), the electrocorticogram (ECoG), or depth electrodes. Many types of task-related information (or features) can be extracted from brain waves, depending on the specific experimental protocol and expected neurophysiological activation pattern. Prominent examples include event-related potentials (ERP) (Blankertz et al., 2011), steady-state evoked potentials (SSEP) (Prueckl and Guger, 2009), event-related (de-)synchronization (Pfurtscheller and Lopes da Silva, 1999), and high-gamma activation (Miller et al., 2009; Kapeller et al., 2018). The latter refers to power changes in frequencies above 50 Hz, which can only be computed from invasively recorded data, such as from ECoG or depth electrodes. Invasive BCIs that operate on high-gamma based features have gained considerable attention and are subject to intensive research. For example, this encompasses real-time passive functional mapping in the course of surgery planning (Ogawa et al., 2014), visual categorization tasks (Kapeller et al., 2018), or the development of BCI prototypes for prosthetic limb, hand, or finger control (Shenoy et al., 2007; Kubánek et al., 2009; Onaran et al., 2011; Yanagisawa et al., 2011; Pistohl et al., 2012; Chestek et al., 2013; Kapeller et al., 2014; Xie et al., 2015; Bleichner et al., 2016; Hotson et al., 2016; Branco et al., 2017; Jiang et al., 2017; Li et al., 2017; Pan et al., 2018).

It is known that high-gamma based invasive BCIs can yield very high classification accuracies, depending on the complexity of the experiment, the electrode location and density, and the processing methods and parameters. For example, Yanagisawa et al. (2011) classified hand movement vs. rest in one patient with an accuracy of 79.6%. Pistohl et al. (2012) achieved an average accuracy of 87.8% over three subjects for two different types of grasp movements. In another study, two high-level hand gestures were correctly identified at an average rate of 95.5% over four subjects (Xie et al., 2015). All these results entailed standard ECoG grids. Performance increases considerably when high-density electrode arrays are employed. Among others, this was shown by Jiang et al. (2017), who achieved 100% accuracy for two subjects in a two-class experiment involving hand gestures.

Three or more different hand gestures can also be identified by high-gamma based BCIs. Yanagisawa et al. (2011) reported 68.3% in one subject for three different hand postures. For the same experiment, Kapeller et al. (2014) published accuracies up to 95.9% averaged over two subjects, and Li et al. (2017) scored on average 80.0% in three subjects. In a similar setup, Xie et al. (2015) obtained correct classification rates of 92.7% over three hand gestures in four subjects on average. Whereas, these experiments were obtained from standard ECoG grids, several studies with hand posture classification were conducted with subjects having high-density grids implanted. Using both high-density and standard electrode arrays, Chestek et al. (2013) conducted experiments to discriminate four hand postures and rest at an accuracy rate of 77.7% on average over three subjects. Using only high-density grids, Pan et al. (2018) reported up to 90% for three different hand gestures over 5 subjects. Involving four different hand gestures, Bleichner et al. (2016) achieved 85.5% accuracy over two subjects, and Branco et al. (2017) attained 85.0% over five subjects.

Many efforts have also gone into decoding individual finger movements. Using standard ECoG grids, Shenoy et al. (2007) achieved an average accuracy of 77.0% for classifying each of the five fingers over six subjects. Kubánek et al. (2009) reported 80.3% over five subjects for the same experiment, whereas Onaran et al. (2011) got 86.3% over three subjects. Four fingers vs. rest were correctly decoded at a rate of 79.3% in three subjects by Chestek et al. (2013). One subject with high-density electrodes implanted achieved 96.5% accuracy for each finger in a study conducted by Hotson et al. (2016).

Table 1 summarizes these results and provides a comprehensive overview of the state of the art.

**Table 1:** State-of-the art summary of hand-motor decoding experiments involving high-gamma based invasive BCIs.

**Reference**	**No. of subjects**	**Electrode spacing^**a**^**	**Frequency bands (Hz)**	**Feature reduction^**b**^**	**Classifier^**c**^**	**Trial alignment**	**Trial length (s)^**d**^**	**Protocol^**e**^**	**Classes^**f**^**	**Mean accuracy (%)**
Shenoy et al., 2007	6	Macro	11 − 40 71 − 100 101 − 150	None	LPM	None	Not reported	Finger	5	77.0
Kubánek et al., 2009	5	Macro	8 − 12 18 − 24 75 − 115 125 − 159 159 − 175	None	LMD	Data glove	[−1.0, +1.0]	Finger	5	80.3
Onaran et al., 2011	3	Macro	65 − 200	CSP	SVM	Data glove	[−0.75,+1.0]	Finger	5	86.3
Yanagisawa et al., 2011	1	Macro	1 − 8 25 − 40 80 − 150	None	SVM	None	n/a (online)	Move RPS	1 + 1 3	79.6 68.3
Pistohl et al., 2012	3	Macro	2 − 6 14 − 46 54 − 114	None	rLDA	Data glove	[−1.0,+0.5]	Grasp	2	87.8
Chestek et al., 2013	3	Mixed	66 − 114	None	NB	Data glove	[−0.5, +1.5]	Gesture Finger	4 + 1 4 + 1	77.7 79.3
Kapeller et al., 2014	2	Macro	60 − 90 110 − 140 160 − 190	FS CSP	LDA	None	[−0.5,+1.5]	RPS	3	83.8 95.8
Xie et al., 2015	4	Macro	Auto	FS	LDA	None	Various	Gesture	2 3	95.5 92.7
Bleichner et al., 2016	2	Micro	70 − 125	FS	PM	Data glove	[−1.0,+2.0]	Gesture	4	85.5
Hotson et al., 2016	1	Micro	72 − 110	FS	LDA	Data glove	[−0.4, +1.0]	Finger	5	96.5
Branco et al., 2017	5	Micro	70 − 125	None	PM	High-gamma	[−1.0,+2.6]	Gesture	4	85.0
Jiang et al., 2017	2	Micro	60 − 200	CSP	LDA	Not reported	[−0.15, +0.35]	Gesture	2	100.0
Li et al., 2017	3	Macro	4 − 12 70 − 135	FS	SVM	None	[±0.0, +0.9]	RPS	3	≈80
Pan et al., 2018	5	Micro	4 − 12 12 − 40 40 − 70 70 − 135 135 − 200	FS	RNN	Data glove	[±0.0, +0.5] [±0.0, +1.2]	RPS	3	≈80 ≈90

a *Macro, standard ECoG grid; Micro, high-density ECoG grid; Mixed, standard and high-density ECoG grids*.

b *CSP, common spatial patterns; FS, algorithm-based or manual channel/feature selection*.

c *LPM, linear programming machine; LMD, linear multivariate decoder; SVM, support vector machine; (r)LDA, (regularized) LDA; NB, naive Bayes; PM, pattern matching; RNN, recurrent neural network*.

d *Specified relative to cue, movement onset, or high-gamma onset (depending on trial alignment)*.

e *Finger, finger movement or tapping; Move, movement vs. rest; RPS, rock-paper-scissors; Gesture, arbitrary hand gestures*.

f *Inclusion of a resting-state class denoted by “+1”*.

A variety of classifiers for both offline and real-time BCIs exist. Besides linear programming machines (Shenoy et al., 2007), Bayesian approaches (Chestek et al., 2013), pattern matching (Bleichner et al., 2016; Branco et al., 2017; Kapeller et al., 2018), neural networks (Pan et al., 2018), and support vector machines (Onaran et al., 2011; Yanagisawa et al., 2011; Li et al., 2017), linear discriminant analysis (LDA) is widely used for both non-invasive and invasive BCI and all types of features (Bostanov, 2004; Scherer et al., 2004; Blankertz et al., 2008, 2011; Hoffmann et al., 2008; Prueckl and Guger, 2009; Onaran et al., 2011; Yanagisawa et al., 2011; Pistohl et al., 2012; Kapeller et al., 2014; Xu et al., 2014; Lotte et al., 2015; Xie et al., 2015; Hotson et al., 2016; Gruenwald et al., 2017a; Jiang et al., 2017; Li et al., 2017). LDA is robust, has low complexity due to linearity and performs well in line with more sophisticated methods (Garrett et al., 2003; Lee et al., 2005; Lotte et al., 2007).

If the dimension of the feature space is high, a spatial filter must be employed to reduce the number of features and to prevent the classifier from overfitting. The most straightforward approach is feature selection, either manual from a-priori data inspection or automatized via statistical algorithms (Kapeller et al., 2014; Xie et al., 2015; Bleichner et al., 2016; Hotson et al., 2016; Li et al., 2017; Pan et al., 2018). Another approach for feature reduction in invasive and non-invasive bandpower-based BCIs is common spatial patterns (CSPs), a linear projection scheme that optimizes class separation within a pre-defined window (Blankertz et al., 2008; Onaran et al., 2011; Wu et al., 2013; Kapeller et al., 2014, 2018; Lotte et al., 2015; Gruenwald et al., 2017a).

To underline the popularity of the aforementioned methods, 5 out of 14 setups as listed in Table 1 utilize LDA while scoring top results, and all feature reduction approaches (9 out of 15) are either selection-based or CSP-based.

Despite their striking advantages, all of the three outlined techniques (LDA, CSP, and feature selection) suffer from substantial drawbacks.

First of all, LDA is inherently static, since it is designed to operate on two multidimensional point clouds. However, the trials of (synchronous) BCIs are usually given as spatiotemporal feature matrices that also contain transient information. This transient information cannot be exploited by LDA in a straightforward manner. Sometimes, it is feasible to vectorize the feature matrices and apply LDA on the resulting vectors. This approach however inflates the dimension of the feature space dramatically. It therefore requires a large amount of trials to maintain statistical robustness, which are only available in particular BCI protocols (such as in P300-based experiments; Hoffmann et al., 2008). If the statistics are too weak for this approach, a regularized version of LDA may be used. In the current context of invasive BCIs for motor control, this approach was followed by Li et al. (2017), whose feature space was spanned by the vectorized power samples from the time × frequency × channel cube. Another attempt to explicitly account for feature transients was pursued by Pan et al. (2018), who employed recursive neural networks. In general, however, LDA is usually employed such that it is applied to the features at a given point in time within the trial that promises to yield good performance. In turn, this creates the challenge of robustly identifying this time point. Furthermore, the features are usually temporally smoothed to enhance performance—the appropriate smoothing level must thus be found empirically as well.

To reduce the dimension of the feature space, feature selection is straightforward and seems to deliver satisfying performance. However, the nature of *selecting* a feature entirely dismisses information in unselected features. Moreover, feature selection is unable to combine joint information from coherent features, leading to redundancy in the final feature set. The search for the optimal set of features may be computationally demanding, if statistically robust results should be obtained. In any case, not only identifying the features themselves, but also determining the appropriate *number* of features is an additional degree of freedom of this method that must be properly taken care of.

In contrast to feature selection, CSPs inherently overcome the two main issues of feature selection, such as information redundancy and feature dismissal. However, finding the optimal location and size of the CSP window may be challenging and requires manual intervention. To the best of our knowledge, no automatized approaches thereto exist. As well, the optimal number of features to be selected for optimal performance needs to be determined in advance.

In view of the shortcomings of state-of-the art methods delineated above, we here present a novel classification method for machine-learning systems with spatiotemporal features in general, and for high-gamma based invasive BCIs for motor control in particular. Our method extends LDA such that it accounts for the time-varying nature of features, we thus name it *time-variant* linear discriminant analysis (TVLDA). Since TVLDA is applied to one trial as a whole, it avoids the need of estimating the optimal time point for classification as was necessary for LDA. We will also describe an intrinsic property of TVLDA that allows for straightforward and powerful feature reduction via principal component analysis (PCA). Additionally, we investigate the impact of a simple time-domain spectral whitening stage during preprocessing. The resulting system is still linear and of low complexity, which enables it for future real-time experiments. We quantitatively assess and compare the performance of our method by means of recordings from 15 subjects with temporarily implanted ECoG electrodes.

## 2. Materials and Methods

### 2.1. Subjects

#### 2.1.1. Original Study

In the original study conducted for this publication, we evaluated data from six epilepsy patients undergoing surgical treatment at Asahikawa Medical University, Asahikawa, Japan. For surgery planning, the patients had a variety of ECoG grids of different types, sizes, and channel counts implanted over the course of several weeks. Besides the standard clinical procedure, all of them volunteered to participate in additional research experiments. The study was approved by the institutional review board of Asahikawa Medical University and received certificate number 245 in June 2012. Written informed consent was obtained from each patient before participating in the study.

Table 2 summarizes the most important data and recordings from the patients, which we subsequently refer to as subjects S1 through S6. Their ages ranged between 17 and 37 years at the day of electrode implantation. S1 and S4 (one third) are female, whereas S2, S3, S5, and S6 are male. S4 is the only left-handed subject. Covered hemispheres are left for S3 and S4 and right for the others.

**Table 2:** Subjects S1–S6 and experiment overview of the original study conducted in Asahikawa, Japan.

**ID**	**Age**	**Gender**	**Handed-ness**	**Covered hemisphere**	**Electrode spacing**^**a**^	**Electrodes total**	**Electrodes selected**	**Coverage motor**^**b**^	**Coverage somatosensory**^**b**^	**Protocol^**c**^**	**Trials per class**
S1	35	Female	Right	Right	Macro	98	20	7–8	5–7	RPS	30
S2	26	Male	Right	Right	Micro	140	60	26–32	19–24	FTPU RPS	40 40
S3	26	Male	Right	Left	Micro	187	60	29–36	22–26	FTPU	20
S4	17	Female	Left	Left	Micro	164	60	29–37	12–17	FTPD FTPU RPS	75 86 65
S5	22	Male	Right	Right	Micro	158	60	5–7	27–34	FTPU RPS	97 76
S6	37	Male	Right	Right	Macro	100	18	7–9	4–7	RPS	60

a *Macro, standard ECoG grid; Micro, high-density ECoG grid*.

b *Estimated number of electrodes, based on Figure 1*.

c *RPS, rock-paper-scissors; FTPD, finger tapping, palm down; FTPU, finger tapping, palm up*.

From the total number of implanted ECoG grids, we only used the ones covering sensorimotor areas for further evaluation. These were standard 20-channel grids (Unique Medical Co., Ltd., Tokyo, Japan; diameter 3 mm, spacing 10 mm, geometry 4 × 5) for S1 and S6, and 60-channel high-density ECoG grids (Unique Medical Co., Ltd.; diameter 1.5 mm, spacing 5 mm, geometry 6 × 10) for the others. Based on MRI and CT scans, we reconstructed a three-dimensional model of the brain via *FreeSurfer* (Martinos Center for Biomedical Imaging, Harvard University) and co-registered electrode locations. Based on this and a functional parcellation of the brain, we roughly estimated the electrode coverage on the primary motor cortex and the somatosensory cortex. At this stage, it turned out that the electrodes of S5 were actually only covering somatosensory areas. Figure 1 provides an overview of the electrode placement.

**Figure 1:**
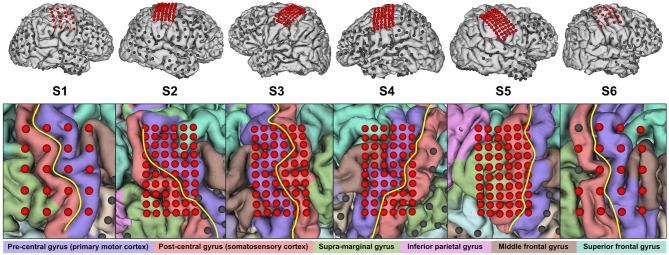
Electrode placement overview. Electrodes reported in Table 2 are highlighted in red. Not all of the remaining electrodes in the top row are visible due to occlusion. In the close-up view, the central sulcus is indicated in yellow and the identified gyri are shaded in respective colors.

#### 2.1.2. Public Dataset

In order to make our analyses reproducible by other researchers, we also evaluated the publicly available *fingerflex* dataset^1^ from Kai Miller.

##### 2.1.2.1. Ethics statement

All patients participated in a purely voluntary manner, after providing informed written consent, under experimental protocols approved by the Institutional Review Board of the University of Washington (no. 12193). All patient data was anonymized according to IRB protocol, in accordance with HIPAA mandate. These data originally appeared in the manuscript *Human Motor Cortical Activity Is Selectively Phase- Entrained on Underlying Rhythms* published in PLoS Computational Biology in 2012 (Miller et al., 2012).

This dataset contains nine subjects, which we integrate as S7 through S15 in this context. A brief summary is given in Table 3. All subjects used implanted platinum arrays (Ad-Tech Medical Instrument Corporation, Wisonsin, USA) with 2.3 mm exposed surface and 10 mm inter-electrode distance. The datasets comprised a variable number of channels, which all seemed to contain good ECoG data. In contrast to the data from our study in Asahikawa, it was difficult to assess the exact coverage of S7–S15; we thus used all channels for further processing. Please see the original publication for more details regarding the exact electrode locations.

**Table 3:** Subjects S7–S15 and experiment overview of the public ECoG dataset (S7–S9 are identical with Subject 1–3 from the BCI Competition IV, respectively).

**ID**	**Patient code**^**a**^	**BCI comp. IV**	**Age**	**Gender**	**Handed-ness**	**Covered hemisphere**	**Electrode spacing^**b**^**	**No. of electrodes**	**Protocol^**c**^**	**Trials per class**
S7	bp	Subject 1	18	Female	Right	Left	Macro	46	FTPU	28
S8	cc	Subject 2	21	Male	Right	Right	Macro	63	FTPU	28
S9	zt	Subject 3	27	Female	Right	Left	Macro	61	FTPU	28
S10	jp		35	Female	Right	Left	Macro	58	FTPU	18
S11	ht		26	Male	Right	Left	Macro	64	FTPU	27
S12	mv		45	Female	Right	Left	Macro	43	FTPU	6
S13	wc		32	Male	Right	Left	Macro	64	FTPU	28
S14	wm		19	Female	Right	Right	Macro	38	FTPU	14
S15	jc		18	Female	Right	Left	Macro	47	FTPU	23

a *As stated in the dataset documentation*.

b *Macro, standard ECoG grid*.

c *FTPU, finger tapping, palm up*.

We recognized that the recordings from S7 to S9 are identical with Subject 1–3 from the BCI Competition IV, respectively, which is another highly popular public ECoG dataset (Tangermann et al., 2012).

### 2.2. Experiments

Table 2 summarizes the conducted experiments, which all relate to hand motor functions at different abstraction levels. The *rock-paper-scissors* (RPS) experiment addresses high-level gestures, whereas the *finger-tapping* experiment aims at decoding individual finger movement. The latter is divided into the two variants *palm down* (FTPD) and *palm up* (FTPU). We will use the terms finger movement and finger tapping interchangeably throughout this publication.

**Figure 2:**
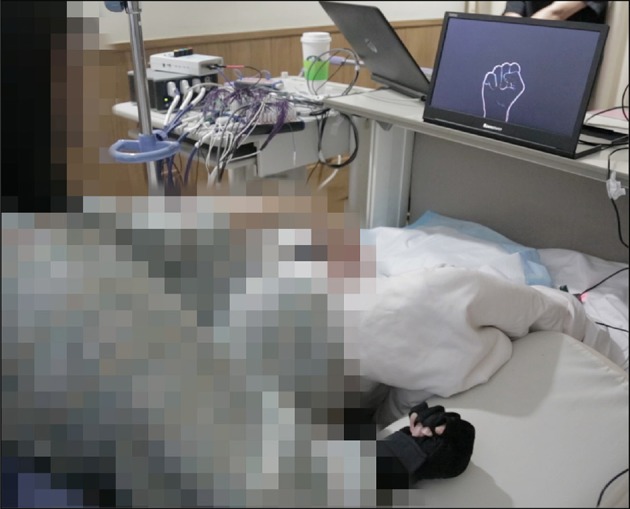
Setup of the rock-paper-scissors experiment.

All experiments were conducted at the bedside of the patient. Before each experiment, the patients received and confirmed all necessary instructions to successfully perform it. The respective tasks were triggered by a visual cue, shown on a computer monitor placed in front of the patient. A data glove (5DT Inc., Florida, USA) was used to capture the hand movements of all subjects. In all experiments, the contralateral hand relative to the implantation site carried out the movements. Figure 2 gives a visual impression of the setup. The experiments conducted with S1–S6 were repeated over the course of several days, depending on the condition and motivation of the subjects.

In the following, we describe the individual experiments more in detail.

#### 2.2.1. Rock-Paper-Scissors

The different hand poses involved in this experiment were inspired by the well-known hand game *rock-paper-scissors* (RPS), constituting a three-class experiment. The visual cues were shown on the screen for one second, interleaved by a scrambled picture distractor of randomized duration between 1.5 and 2.5 seconds. The subjects were instructed to form the requested gesture with their hand once the stimulus appeared, and to return into a relaxed hand position once the distractor showed up. One run included 20 trials per class. The rock-paper-scissors experiment was only conducted with S1–S6.

#### 2.2.2. Finger Tapping, Palm Down and Palm Up

##### 2.2.2.1. Original study (S1–S6)

Here, the subjects were asked to perform two taps with the finger indicated on the screen for one second. Between the cues, a scrambled picture was shown for a randomized duration between 1.7 and 2.5 seconds, indicating that the subject should stay at rest. In the easier version, the palm-up version (FTPU), the subjects executed two repeated finger flexions, whereas in the palm-down version (FTPD), the subjects performed actual taps on a solid, planar surface. One run of this five-class experiment consisted of 10 trials per class. The palm-down version turned out to require a certain level of fine motor skills that was not present in all subjects, so only S4 completed it successfully.

Due to misunderstanding of the task instructions, S3 executed this experiment differently: instead of executing two discrete taps, he kept flexing the finger until a new instruction showed up on the screen. As described below, this required some additional processing steps to obtain usable data.

##### 2.2.2.2. Public dataset (S7–S15)

As described in Miller et al. (2012), the subjects were cued with a word shown on a bedside monitor, indicating which finger to move. Each task lasted for two seconds, during which the subjects typically performed between two and five repeated finger flexions. A blank screen, shown for another two seconds, was interleaved between each task as a resting trial. Only the palm-up variant of the experiment was performed.

### 2.3. Data Acquisition

#### 2.3.1. Original Study (S1–S6)

We captured the raw ECoG data with the *g.HIamp* biosignal amplifier (g.tec medical engineering GmbH, Austria) and used *Simulink* (The MathWorks, Inc., Massachusetts, USA) as the recording environment. Depending on the overall number of channels, we set the sampling rate to either 1.2 or 2.4 kHz. We used the *g.HIsys Highspeed Online Processing* toolbox (g.tec medical engineering GmbH) for the stimulus presentation and synchronous data acquisition and storage. The recorded data were saved on a hard drive and re-processed offline in *MATLAB* (The MathWorks, Inc.) for this study as described in this section.

#### 2.3.2. Public Dataset (S7–S15)

As communicated by Miller et al. (2012), the ECoG data were recorded with the *Synamps 2* biosignal amplifier (Compumedics Neuroscan, North Carolina, USA) at a sampling rate of 1 kHz and internal bandpass-filter from 0.3 to 200 Hz. The general-purpose software environment *BCI2000* was used for stimulus presentation and synchronous data acquisition.

### 2.4. Preprocessing and Feature Extraction

This subsection closely follows the concept of Gruenwald et al. (2017b), which outlines optimal bandpower estimation for real-time BCIs. If not otherwise mentioned, we processed data from all subjects regardless of their origin in the exactly same manner.

After excluding channels that were notably bad due to high impedance, we re-referenced the data by the common average. After that, a notch-filter cascade (recursive 6th-order Butterworth, bandwidth: 5 Hz) up to the 6th harmonic was used to remove interference peaks from the spectrum at integer multiples of the power line frequency.

Next, an optional spectral whitening filter (Oppenheim and Schafer, 2010) was applied to each channel. While the concept of whitening (or spectral equalization) is frequently used in time-frequency analysis (Miller et al., 2009; Yanagisawa et al., 2011; Pistohl et al., 2012), it is less known that it can also be performed in time domain by a simple finite-impulse response filter. This enables whitening for real-time applications, where time-frequency transformation is not an option.

The underlying principle of a whitening filter is that the input signal can be modeled as a *P*th-order autoregressive (AR) process, e.g.,

(1) $$\begin{array}{l} \overset{P}{\underset{p\;=\;0}{\sum }}a_{p}y\left[n-p\right]=v\left[n\right] \end{array}$$

with $v\left[n\right]∽N\left(0,\sigma_{v}^{2}\right)\text{i.i.d.}$ being a zero-mean white Gaussian noise with variance $\sigma_{v}^{2}$. In this publication, we use the tilde notation to link a random variable with its particular distribution and the term i.i.d. to indicate independent and identically distributed samples. The AR coefficients *a*_*p*_ can be determined by the *Yule-Walker equations* that are applied to a sufficiently long signal fragment of *y*[*n*] (e.g., a few seconds). Equation 1 can now be seen as a linear time-invariant filter with impulse response *a*_*p*_:

(2) $$\begin{array}{l} ȳ\left[n\right]=\overset{P}{\underset{p\;=\;0}{\sum }}a_{p}y\left[n-p\right]. \end{array}$$

It is intuitive that the filter output *ȳ*[*n*] resembles the white noise *v*[*n*] and therefore has a flat spectrum.

**Figure 3:**
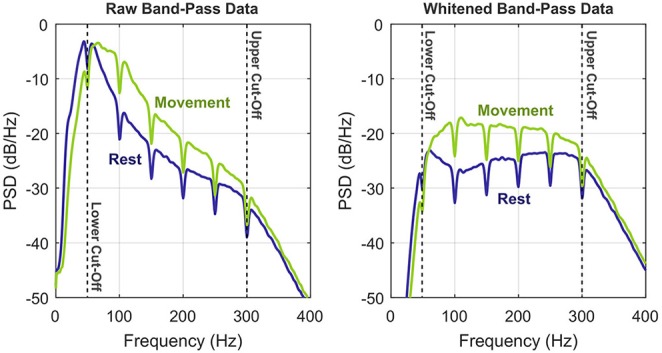
Illustration of the whitening procedure by means of power spectral densities of the preprocessed bandpass signals (S6, RPS, exemplary channel). To illustrate the benefits of whitening, the two conditions *rest* vs. *movement* (any class) are shown separately. Upper and lower corner frequencies of the bandpass filter are indicated by the vertical dashed lines.

The positive effect of whitening on the signal-to-noise ratio of ECoG bandpower features was anticipated previously (Gruenwald et al., 2017b). Figure 3 provides an illustration, where the dynamic range of the signal within the cut-off frequencies can be roughly estimated to 25 dB. Whitening equalizes the pronounced 1/*f*-shape of the spectrum, which balances the frequency-specific contributions to the overall bandpower and thus increases signal fidelity.

Since an ECoG spectrum is rather smooth in absence of interference peaks, the filter order can be low. In practice, we found a 10th-order whitening filter sufficient.

After the optional whitening stage, we band-passed the signal (recursive 6th-order Butterworth) to our high-gamma frequency band of interest. We assessed several bands with respect to classification performance, and finally chose 50 to 300 Hz as our target. This may seem inappropriate in view of the fact that the data of S7–S15 was pre-filtered by a bandpass between 0.3 and 200 Hz. However, we observed that the whitening procedure was able to recover high-gamma components well above 200 Hz.

Given the bandpass signals, we then estimated the bandpower via a sliding variance window of 50 ms length, without overlap. A log-transform was appended, to improve signal stationarity and Gaussianity.

Then, the data were triggered, i.e., cut into signal fragments for each trial and class. Since S3 and S7–S15 exhibited a large movement onset jitter, we applied a trial-based correction. To this end, we used the signals captured by the data glove for aligning the individual trials of S3. Likewise, we corrected the onset jitter of S7 to S15 by a movement trigger already contained in the data. For the other subjects (S1, S2, and S4–S6), no explicit trial alignment was performed, since the onset jitter was already small enough for good classification results. However, we compensated for the systematic reaction and execution latency by shifting the grand average high-gamma onset to the center of the trial to guarantee symmetry.

We set our trial length to 0.75 seconds pre- and post-onset, respectively. Trials that were contaminated with pathological brain activity (such as inter-ictal spiking) were removed. No further trial exclusion was performed.

At this point, it is reasonable to establish a mathematical model that facilitates subsequent methodological derivations. To this end, we refer to the number of samples and channels as *N*_S_ and *N*_Ch_, respectively. The preprocessed and triggered data then constitute spatiotemporal feature matrices ${\text{Y}}_{\text{c}}^{\left(i\right)}\in {\mathbb{R}}^{N_{\text{S}}\times N_{\text{Ch}}}$ for trials *i* and classes c. Both trials and classes are expected to stem from a pool of *N*_T_ trials and *N*_C_ classes, respectively, e.g., *T* = {1, …, *N*_T_} and C = {A, B, C, … } with |C| = *N*_C_. For the typical machine-learning scenario, we are further partitioning the set of trials into a training set *T*_Train_ (with known class labels) and a test set *T*_Test_ (with unknown class labels), which are disjoint. Formally, these sets can be expressed as

(3) $$\begin{array}{l} Y_{\text{Train}}=\{{\text{Y}}_{\text{c}}^{\left(i\right)}|\text{c}\in C,i\in T_{\text{Train}}\} \end{array}$$

(4) $$\begin{array}{l} Y_{\text{Test}}=\{{\text{Y}}_{\text{c}}^{\left(i\right)}|\text{c}\in C,i\in T_{\text{Test}}\}. \end{array}$$

Toward mathematical tractability, we decompose ${\text{Y}}_{\text{c}}^{\left(i\right)}$ into row vectors ${\text{y}}_{\text{c}}^{\left(i\right)}\left[n\right]$ with discrete-time index *n*:

(5) $$\begin{array}{l} ({\text{Y}}_{\text{c}}^{\left(i\right)}{)}_{n,\cdot }={\text{y}}_{\text{c}}^{\left(i\right)}\left[n\right],\;n\in \left\{0,1,\ldots ,N_{\text{S}}-1\right\}. \end{array}$$

### 2.5. Feature Reduction (Standard)

The number of recorded channels may be high, particularly in ECoG experiments. This increases the computational demands and the risk of classifier overfitting. Consequently, a feature projection or selection stage usually precedes the classifier. Especially for ECoG, this can decrease dimensionality tremendously without losing information, since (1) only a limited amount of channels significantly contributes to class separation and (2) correlation across contributing channels may be high. Mathematically, this feature projection is implemented by a generic matrix $\text{P}\in {\mathbb{R}}^{N_{\text{Ch}}\times N_{\text{F}}}$ with *N*_F_ ≪ *N*_Ch_, such that

(6) $$\begin{array}{l} {\text{x}}_{\text{c}}^{\left(i\right)}\left[n\right]={\text{y}}_{\text{c}}^{\left(i\right)}\left[n\right]\text{P}. \end{array}$$

Following a likewise decomposition as in (5), we denote the contracted spatiotemporal feature matrices by ${\text{X}}_{\text{c}}^{\left(i\right)}\in {\mathbb{R}}^{N_{\text{S}}\times N_{\text{F}}}$ and write for the training and test sets

(7) $$\begin{array}{l} X_{\text{Train}}=\{{\text{X}}_{\text{c}}^{\left(i\right)}|\text{c}\in C,i\in T_{\text{Train}}\} \end{array}$$

(8) $$\begin{array}{l} X_{\text{Test}}=\{{\text{X}}_{\text{c}}^{\left(i\right)}|\text{c}\in C,i\in T_{\text{Test}}\}. \end{array}$$

The subsections below describe strategies how to populate the projection matrix **P**.

#### 2.5.1. Common Spatial Patterns

Common spatial patterns (CSPs) are the de-facto standard for dimension reduction in EEG signal processing (Blankertz et al., 2008; Lotte et al., 2015) and are also popular in ECoG signal processing (Onaran et al., 2011; Kapeller et al., 2014; Xie et al., 2015; Jiang et al., 2017). This approach expects multivariate distributions of two classes A and B with covariances **Σ**_A_ and **Σ**_B_, respectively. The CSP transformation matrix then simultaneously diagonalizes both **Σ**_A_ and **Σ**_B_, where the element-wise ratio along the diagonals is strictly monotonic. Consequently, the first and the last CSP component maximize the variance for one class, while minimizing it for the other. Additional CSP components further contribute to this.

In the given context, CSPs operate on the triggered bandpass data within a pre-defined window, i.e., *before* power computation. For all datasets, we have located the peak of the grand high-gamma activation over trials and classes, and centered the CSP window about this peak. We set the window length to 0.3 seconds, since this yielded the best classification results. Denoting the CSP transformation by $\text{R}\in {\mathbb{N}}^{N_{\text{Ch}}\times N_{\text{Ch}}}$, the projection matrix **P**_CSP_ is then column-wise populated with the first ⌈*N*_F_/2⌉ and the last ⌊*N*_F_/2⌋ columns of **R**. As will be discussed in section 2.8, we computed CSPs pairwise for each binary classification in a multi-class scenario.

#### 2.5.2. Feature Selection

Another common approach to reduce the dimensionality is a discrete feature (or channel) selection process. While the individual implementations differ considerably, feature selection is heavily used in the ECoG community (Kapeller et al., 2014; Xie et al., 2015; Bleichner et al., 2016; Hotson et al., 2016; Li et al., 2017; Pan et al., 2018).

Here, we use a straightforward approach for feature selection. First, we compute an activation score for each class and channel, which is the trial-averaged relative band-power increase from baseline (before high-gamma onset) to activation (after high-gamma onset). For each pair of classes, we then calculate the absolute difference of this activation score for each channel and sort the result in descending order. This way, the channels exhibiting the largest high-gamma activation *difference* for the two classes are ranked top. Consequently, the projection matrix **P**_FS_ (which is more a *selection* matrix now) is established such that its *N*_F_ columns logically index the first *N*_F_ channels in the given ranking, respectively.

### 2.6. Classification

We now assume that, for each class, the feature matrices ${\text{X}}_{\text{c}}^{\left(i\right)}$ comprise a unique underlying activation pattern that is identical over trials. However, each repetition is subject to noise, most prominently from imperfect task execution and the uncertainty of feature estimation. We thus employ a multivariate Gaussian distribution to describe these components as follows:

(9) $$\begin{array}{l} {\text{x}}_{\text{c}}^{\left(i\right)}\left[n\right]∽N\left(\mu_{\text{c}}\left[n\right],\Sigma_{\text{c}}\left[n\right]\right)\text{i.i.d.} \end{array}$$

In general, $\mu_{\text{c}}\left[n\right]\in {\mathbb{R}}^{1\times N_{\text{F}}}$ and $\Sigma_{\text{c}}\left[n\right]\in {\mathbb{R}}^{N_{\text{F}}\times N_{\text{F}}}$ are not known.

The independence constraint is expected to hold over samples *n*, trials *i* and classes c. While this requirement is intuitively hold over trials and classes, in fact it may be violated over samples. We have shown in Gruenwald et al. (2017b) that the signal processing pipeline yields high-gamma features with estimation noise that can be considered white; however, imperfect trial execution may impose temporally correlated noise on the data. We will also address this issue in section 4.

#### 2.6.1. Linear Discriminant Analysis

A standard tool to separate features of two classes is linear discriminant analysis (LDA). In a nutshell, LDA expects multivariate Gaussian distributions from two classes A and B and finds a projection vector that simultaneously maximizes the mean distance whilst minimizing the individual variances of the projected populations (Bishop, 2006). LDA-based classifiers are optimal in the maximum-likelihood sense if the two distributions are homoscedastic.

For convenience and if applicable, we hereafter use the generic class label c ∈ {A, B} to denote either of the two classes. In case we know the class associated with a particular variable, we denote this by subscript notation.

A common approach to classify spatiotemporal features with LDA is the training of several LDA instances over time and selecting the classifier which yields best performance. Thus, after introducing the well-known difference of means and pooled covariance matrix

(10) $$\Delta \mu [n]=\mu_{\text{B}}[n]-\mu_{\text{A}}[n]$$

(11) $$\begin{array}{l} \Sigma \left[n\right]=\frac{1}{2}(\Sigma_{\text{A}}\left[n\right]+\Sigma_{\text{B}}\left[n\right]), \end{array}$$

the standard LDA projection vector equates to

(12) $$\text{w}[n]=\Delta \mu [n]\Sigma^{-1}[n].$$

Given an arbitrary input **x**^(*i*)^[*n*], the symmetric LDA score *p*^(*i*)^[*n*] is computed as

(13) $$\begin{array}{l} p^{\left(i\right)}\left[n\right]=\text{w}\left[n\right]{\text{x}}^{\left(i\right)}{\left[n\right]}^{\text{T}}-d\left[n\right], \end{array}$$

where the superscript (·)^T^ denotes matrix transposition and the offset

(14) $$\begin{array}{l} d\left[n\right]=\frac{1}{2}\text{w}\left[n\right]{\left(\mu_{\text{A}}\left[n\right]+\mu_{\text{B}}\left[n\right]\right)}^{\text{T}} \end{array}$$

centers the two projected class populations about zero. This can be verified by equating the means $\mu_{p_{\text{c}}}\left[n\right]=\text{E}\left\{p_{\text{c}}^{\left(i\right)}\left[n\right]\right\}$ via Equations (13) and (14), where E{·} denotes the expectation operator over trials. It is now evident that μ_*p*_A__[*n*] = −μ_*p*_B__[*n*], since

(15) $$\mu_{p_{\text{A}}}[n]=-\frac{1}{2}\text{w}[n]\Delta \mu {\left[n\right]}^{\text{T}}$$

(16) $$\mu_{p_{\text{B}}}[n]=+\frac{1}{2}\text{w}[n]\Delta \mu {\left[n\right]}^{\text{T}}.$$

##### 2.6.1.1. Training

There are different approaches to apply an LDA classifier to spatiotemporal features. The most common strategy is to smooth the features over time, train the LDA classifier for each point in time, and then select the one which gives best performance. In the present context, best performance for LDA was achieved if the features within a trial were symmetrically smoothed by three samples in each direction. Toward the decision which classification time point to use, we investigated several options. Most robust results were obtained by adaptively selecting the time point of maximum high-gamma activation over all classes and trials. We subsequently denote this time point as *n*_LDA_.

Summarizing the LDA training procedure, the sample means ${\hat{\mu }}_{\text{c}}$[*n*_LDA_] and covariances ${\hat{\Sigma }}_{\text{c}}$[*n*_LDA_] are computed first, given labeled training data ${\text{X}}_{\text{c}}^{\left(i\right)}\in X_{\text{Train}}$. Via Equations (10), (11), (12), and (14), the set $\left\{\hat{\text{w}}\left[n_{\text{LDA}}\right],\hat{d}\left[n_{\text{LDA}}\right]\right\}$ then constitutes the LDA classifier.

##### 2.6.1.2. Test

Given a test trial ${\text{X}}^{\left(i\right)}\in X_{\text{Test}}$ and $\left\{\hat{\text{w}}\left[n_{\text{LDA}}\right],\hat{d}\left[n_{\text{LDA}}\right]\right\}$ as the classifier, the LDA score ${\hat{p}}^{\left(i\right)}$ is simply computed analogously to (13):

(17) $$\begin{array}{l} {\hat{p}}^{\left(i\right)}=\hat{\text{w}}\left[n_{\text{LDA}}\right]{\text{x}}^{\left(i\right)}{\left[n_{\text{LDA}}\right]}^{\text{T}}-\hat{d}\left[n_{\text{LDA}}\right]. \end{array}$$

Since the two classes in question lead to LDA scores symmetric about zero, the natural threshold for classification is zero as well:

(18) $${\hat{c}}_{\text{LDA}}^{\left(i\right)}=\{\begin{array}{ll} \text{A} & {\hat{p}}^{\left(i\right)}<0\\ \text{B} & {\hat{p}}^{\left(i\right)}\geq 0 \end{array}.$$

#### 2.6.2. Regularized Linear Discriminant Analysis

Computing the LDA weight vector requires the inversion of the pooled covariance matrix. This can become numerically unstable if the number of samples is not much larger than the feature dimensionality. To overcome this problem, a regularized LDA (rLDA) can be used where only the main diagonal of the sample covariance matrices is accounted for (also known as *shrinking*). Since this allows stable inversion even in high-dimensional feature space, rLDA is particularly appealing when applied to vectorized features ${\bar{\text{x}}}_{\text{c}}^{\left(i\right)}\in {\mathbb{R}}^{1\times N_{\text{S}}N_{\text{F}}}$, such that

(19) $${\bar{x}}_{\text{c}}^{\left(i\right)}=\left[x_{\text{c}}^{\left(i\right)}[0],x_{\text{c}}^{\left(i\right)}[1],\ldots ,x_{\text{c}}^{\left(i\right)}[N_{S}-1]\right]$$

(20) $$\begin{array}{l} ∽N\left({\bar{\mu }}_{\text{c}},{\bar{\Sigma }}_{\text{c}}\right)\text{ i.i.d.} \end{array}$$

to account for all spatiotemporal information at once.

##### 2.6.2.1. Training

Training the rLDA classifier is straightforward. After computing sample means and sample covariance matrices from the vectorized training data, the off-diagonal elements of the sample covariance matrices are set to zero. Equations (10), (11), (12), and (14) yield the rLDA classifier $\left\{{\hat{w}}^{*},{\hat{d}}^{*}\right\}$. Note that the temporal index *n* has now vanished.

##### 2.6.2.2. Test

Applying the rLDA classifier $\left\{{\hat{w}}^{*},{\hat{d}}^{*}\right\}$ to test data follows analogously to section 2.6.1.2.

#### 2.6.3. Time-Variant Linear Discriminant Analysis

The major improvement of time-variant linear discriminant analysis (TVLDA) over standard LDA is that it utilizes information of *all* individually trained LDA classifiers over the whole trial, which makes it inherently time-*variant*. To derive the concept of TVLDA mathematically, we first interpret *p*^(*i*)^[*n*] (13) as an *N*_S_-dimensional vector:

(21) $$\begin{array}{l} {\text{p}}^{\left(i\right)}=[p^{\left(i\right)}\left[0\right],p^{\left(i\right)}\left[1\right],\ldots ,p^{\left(i\right)}\left[N_{\text{S}}-1\right]{]}^{\text{T}}. \end{array}$$

In this notation, each class establishes the multivariate Gaussian distribution

(22) $$\begin{array}{l} {\text{p}}_{\text{c}}^{\left(i\right)}∽N(\mu_{{\text{p}}_{\text{c}}},\Sigma_{{\text{p}}_{\text{c}}})\text{ i.i.d.} \end{array}$$

with means $\mu_{{\text{p}}_{\text{c}}}\in {\mathbb{R}}^{N_{\text{S}}\times 1}$ and covariances $\Sigma_{{\text{p}}_{\text{c}}}\in {\mathbb{R}}^{N_{\text{S}}\times N_{\text{S}}}$ equating to

(23) $$\begin{array}{l} \mu_{{\text{p}}_{\text{c}}}=[\mu_{p_{\text{c}}}\left[0\right],\mu_{p_{\text{c}}}\left[1\right],\ldots ,\mu_{p_{\text{c}}}\left[N_{\text{S}}-1\right]{]}^{\text{T}} \end{array}$$

(24) $$\begin{array}{l} \Sigma_{{\text{p}}_{\text{c}}}=\mathrm{diag}\left\{\left[\sigma_{p_{\text{c}}}^{2}\left[0\right],\sigma_{p_{\text{c}}}^{2}\left[1\right],\ldots ,\sigma_{p_{\text{c}}}^{2}\left[N_{\text{S}}-1\right]\right]\right\}. \end{array}$$

The assumed temporal independence of the feature noise (cf. (9)) implicates the fact that **Σ**_**p**_c__ must be diagonal. The elements of **μ**_**p**_c__ are given by Equations (15) and (16), and the elements of **Σ**_**p**_c__ are obtained after short calculus as

(25) $$\begin{array}{l} \sigma_{p_{\text{c}}}^{2}\left[n\right]=\text{w}\left[n\right]\Sigma_{\text{c}}\left[n\right]\text{w}{\left[n\right]}^{\text{T}}. \end{array}$$

We now want to separate the two class populations {${\text{p}}_{\text{A}}^{\left(i\right)}$} and {${\text{p}}_{\text{B}}^{\left(i\right)}$} again in the LDA-sense. Consequently, the difference of means and pooled covariance are given as

(26) $$\Delta \mu_{p}=\mu_{p_{\text{B}}}-\mu_{p_{\text{A}}}$$

(27) $$\begin{array}{l} \Sigma_{\text{p}}=\frac{1}{2}(\Sigma_{{\text{p}}_{\text{A}}}+\Sigma_{{\text{p}}_{\text{B}}}). \end{array}$$

To find an expression for the LDA projection vector $\Sigma_{p}^{-1}\Delta \mu_{p}$, we trace back Equations (23) and (24), Equations (15), (16), and (25), and Equations (11) and (12), to finally arrive at the elegant result

(28) $$\Sigma_{p}^{-1}\Delta \mu_{p}=1.$$

In other words, the overall *TVLDA score*, denoted by *z*^(*i*)^, is simply the sum of all intermediate LDA scores (13):

(29) $$\begin{array}{l} z^{\left(i\right)}=\overset{N_{\text{S}}-1}{\underset{n=0}{\sum }}p^{\left(i\right)}\left[n\right]. \end{array}$$

It is evident that $\mu_{p_{\text{B}}}[n]-\mu_{p_{\text{A}}}[n]=\Delta \mu [n]\Sigma^{-1}[n]\Delta \mu [n{\text{]}}^{\text{T}}$, which can be shown by inserting (12) into Equations (15) and (16). Consequently, the difference of the expected TVLDA scores yields via (29)

(30) $$\text{E}\left\{z_{\text{B}}^{\left(i\right)}\right\}-\text{E}\left\{z_{\text{A}}^{\left(i\right)}\right\}=\sum_{n=0}^{N_{S}-1}\Delta \mu [n]\Sigma^{-1}[n]\Delta \mu {\left[n\right]}^{\text{T}},$$

which is the accumulated Kullback-Leibler divergence of the classes A and B under the homoscedasticity assumption of the (TV)LDA.

##### 2.6.3.1. Training

From the training data ${\text{X}}_{\text{c}}^{\left(i\right)}\in X_{\text{Train}}$, the TVLDA parameters $\left\{\hat{\text{w}}\left[n\right],\hat{d}\left[n\right]\right\}$ are computed based on the sample means ${\hat{\mu }}_{\text{c}}\left[n\right]$ and covariances ${\hat{\Sigma }}_{\text{c}}[n]$ via Equations (10), (11), (12), and (14).

##### 2.6.3.2. Test

For a test trial ${\text{X}}^{\left(i\right)}\in X_{\text{Test}}$ and a set of TVLDA parameters $\left\{\hat{\text{w}}\left[n\right],\hat{d}\left[n\right]\right\}$, the TVLDA score ẑ^(*i*)^ follows according to Equations (29) and (13):

(31) $$\begin{array}{l} {ẑ}^{\left(i\right)}=\overset{N_{\text{S}}-1}{\underset{n=0}{\sum }}\hat{\text{w}}\left[n\right]{\text{x}}^{\left(i\right)}{\left[n\right]}^{\text{T}}-\hat{d}\left[n\right]. \end{array}$$

Evidently, also the TVLDA score is symmetric about zero, which leads to the classification scheme

(32) $${\hat{\text{c}}}_{\text{TVLDA}}^{\left(i\right)}=\{\begin{array}{ll} \text{A} & {\hat{z}}^{\left(i\right)}<0\\ \text{B} & {\hat{z}}^{\left(i\right)}\geq 0 \end{array}.$$

### 2.7. Feature Reduction (TVLDA-Specific)

We here resume section 2.5 by proposing a novel feature dimensionality reduction approach that is intrinsic to TVLDA, based on principal component analysis (PCA).

We can interpret the time-variant TVLDA weight vector as a spatiotemporal weight matrix $\text{W}\in {\mathbb{R}}^{N_{\text{S}}\times N_{\text{F}}}$, whose rows are given by **w**[*n*]:

(33) $$\begin{array}{l} (\text{w}{)}_{n,\cdot }=\text{w}\left[n\right]. \end{array}$$

Motivated by the nature of PCA, we now restrict the generic transformation matrix **P** to be orthogonal, i.e., $\text{P}\in {\mathbb{R}}^{N_{\text{Ch}}\times N_{\text{F}}}$ with *N*_F_ = *N*_Ch_ and **P**^−1^ = **P**^T^. Consequently, any transformation of ${\text{y}}_{\text{c}}^{\left(i\right)}\left[n\right]$ by **P** transparently affects the TVLDA weight matrices **W**_**x**_ and **W**_**y**_:

(34) $$\begin{array}{l} {\text{x}}_{\text{c}}^{\left(i\right)}\left[n\right]={\text{y}}_{\text{c}}^{\left(i\right)}\left[n\right]\text{P}\Rightarrow {\text{W}}_{\text{x}}={\text{W}}_{\text{y}}\text{P}, \end{array}$$

where the subscripts indicate which variable **W** is associated with. This relationship can be shown by substituting the projection scheme into the computation of the weight vector (12) via Equations (10) and (11).

The idea now is to find **P**, such that the weights in **W**_**y**_ are compressed into very few columns of **W**_**x**_. Only these columns of **W**_**x**_ are then kept, leading to an effective reduction in dimensionality.

The standard solution to this problem is PCA, which we implement as a singular value decomposition (SVD) of **W**_**y**_. In short, we factorize ${\text{W}}_{\text{y}}=\text{U}\text{S}{\text{V}}^{\text{T}}$ where $\text{U}\in {\mathbb{R}}^{N_{\text{S}}\times N_{\text{S}}}$ and $\text{V}\in {\mathbb{R}}^{N_{\text{Ch}}\times N_{\text{Ch}}}$ are orthogonal matrices, and $\text{S}\in {\mathbb{R}}^{N_{\text{S}}\times N_{\text{Ch}}}$ is a matrix with zeros, except for the non-negative, decreasing singular values on the diagonal. The desired scores in the principal-component space of **W**_**x**_ are now given by the product **US**, such that we require

(35) $${\text{W}}_{\text{x}}=\text{US}{\text{V}}^{\text{T}}\text{P}\overset{!}{=}\text{US}$$

and obtain simply

(36) $$\begin{array}{l} \text{P}=\text{V}. \end{array}$$

**Figure 4:**
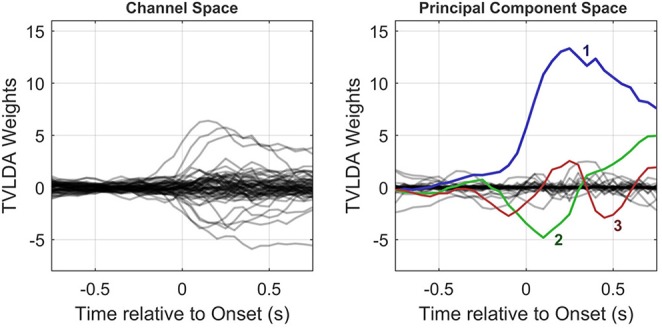
Column-wise visualization of the original and PCA-transformed spatiotemporal weight matrices ***W*_*y*_ (left)** and ***W*_*x*_ (right)**, respectively. As illustrated in the right subplot, only few principal components with large amplitudes remain. This allows for substantial dimension reduction, as detailed in the text.

Since **V** establishes an orthonormal projection, which can be seen as a rotation in high-dimensional space, all information is preserved. The principal components are ordered by their impact, so the projection matrix **P**_PCA_ is simply populated by the first *N*_F_ columns of **V**. Figure 4 provides an example of the PCA-based feature reduction method.

Importantly, the number of channels may be too high to yield invertible covariance matrices (i.e., *N*_Ch_ ≫ *N*_T_). Even if the covariance matrices are nonsingular, their inversion may be numerically unstable. To find a robust PCA decomposition and unless many more trials than channels are available, we therefore recommend smoothing the sample means and covariances over time before computing the weight matrix **W**_**y**_ that is subject to the SVD. In our case, we used bidirectional averaging of two samples in each direction to obtain the best results. For datasets comprising many trials, this bidirectional averaging did not impair results, and hence we recommend using it whenever applicable.

The number of trials may be extremely low, and thus even temporal averaging does not yield a usable PCA decomposition. In this case—and only in this case—we suggest adding a certain level of regularization to PCA: here, the off-diagonal elements of the TVLDA sample covariances are weighted with a factor between 0 and 1, where 0 is identical to complete diagonalization. We have observed that setting this factor to 0.75 (which equals a regularization of 25%) can substantially improve results, especially for datasets with particularly low trial count and a small number of channels capturing task-related activation. We will address this issue further in section 4.

Note that the proposed temporal averaging and regularization only apply for computing the initial **W**_**y**_, but not for **W**_**x**_ after transformation.

### 2.8. Multi-Class Extension

So far, we have only addressed binary classification problems. Since many experiments entail more than two classes, the decision rules defined in Equations (18) and (32) must be extended. We do so by employing a one-vs.-one classification scheme. Consequently, each class is tested against each other class, yielding *N*_C_(*N*_C_ − 1)/2 binary classification results. It would be straightforward to implement a voting approach that elects the winner based on the most votes; however, this approach would lead to frequent ties. Moreover, the quantitative information in the (TV)LDA scores would be lost. We thus propose to use a min-max approach for multi-class discrimination. First, we refer to the (TV)LDA score for class c_*p*_ vs. c_*q*_ as ${ẑ}_{{\text{c}}_{p}{\text{c}}_{q}}^{\left(i\right)}$ (c_*p*_, c_*q*_ ∈ C, c_*p*_ ≠ c_*q*_). The smaller this value gets, the more certain (TV)LDA is that trial *i* belongs to class c_*p*_ rather than to class c_*q*_. Taking the worst score over all classes (i.e., the maximum of ${ẑ}_{{\text{c}}_{p}{\text{c}}_{q}}^{\left(i\right)}$ over all *q*) then indicates how likely it is that trial *i* stems from class c_*p*_, relative to all other classes (the lower the more likely). Finally, the class that minimizes this score is elected:

(37) $$\begin{array}{l} {\hat{c}}_{\text{(TV)LDA}}^{\left(i\right)}=\underset{c_{p}}{\text{arg}\;\text{min}}\{\underset{p\neq q}{\max}\{{\hat{z}}_{{\text{c}}_{p}{\text{c}}_{q}}^{\left(i\right)}\}\} \end{array}$$

Evidently, the feature-reduction techniques discussed in sections 2.5 and 2.7 follow this one-vs.-one scheme as well.

### 2.9. Performance Evaluation

Here, we describe our framework for performance evaluation.

#### 2.9.1. Cross-Validation

We performed 20 repetitions of a randomized 10 × 10 cross-validation to assess the expected performance of the system. All components (such as feature reduction and classification) were subject to this cross-validation to ensure that testing was done on completely unseen data.

#### 2.9.2. Assessed Method Variants

In this publication, we mainly want to investigate the potentials of our proposed improvements, such as (1) spectral whitening, (2) PCA-based feature reduction instead of CSP and feature selection, and (3) TVLDA instead of LDA or rLDA. To this end, we identified seven method variants (or simply methods) that logically follow this path: for LDA with CSP and feature selection, we investigate the effect of whitening. Then, for whitened data, we incorporate rLDA and PCA as a feature reduction technique for LDA. Finally, for whitened data and PCA-based feature reduction, LDA is switched to TVLDA to arrive at the complete set of proposed improvements.

#### 2.9.3. Performance Quantification

We quantify the performance of the respective methods by means of accuracy rates (or simply accuracies). This is the true positive rate, defined as the ratio between correctly classified trials and total number of trials, averaged over all classes. Since our evaluation framework is of statistical nature, a rigorous comparison between methods by means of accuracies is inappropriate. To resolve this, we here define a margin, within which we consider two methods to perform equal. Intuitively, we set this margin to 1/*N*_T_ [%], since this represents the accuracy range that relates to one trial per class. This in turn is the actual quantization level of the respective dataset, and we hereafter refer to it as the *quantization margin*.

To facilitate interpretation and comparison further, we also introduce the term *representative accuracy*. The representative accuracy is an acceptable trade-off between classification accuracy and number of features needed. Since, at some point, increasing *N*_F_ may only marginally contribute to better performance, we chose the smallest *N*_F_ whose corresponding (i.e., representative) accuracy still lies within the quantization margin of the best result.

## 3. Results

In this section, we present the results of the classifier performance evaluation. For the most comprehensive comparison, we included the number of features *N*_F_ from 1 to 15 and evaluated the accuracies for each method variant and dataset.

**Figure 5:**
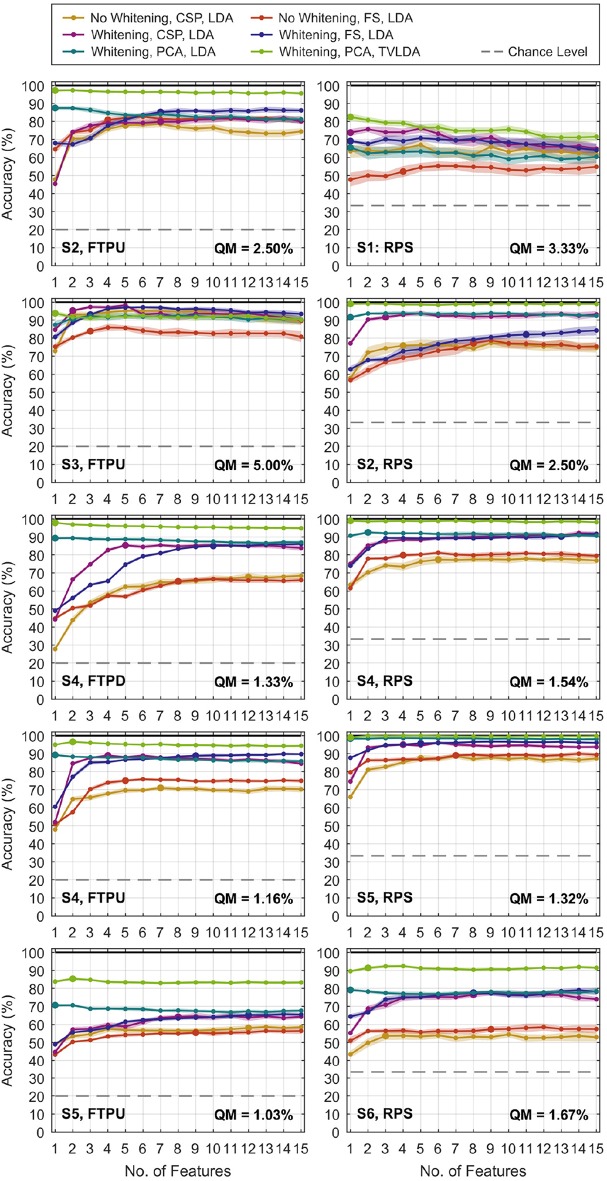
Classification accuracies versus number of features *N*_F_ for selected finger-tapping **(left)** and rock-paper-scissors **(right)** datasets. Results for rLDA are not shown since *N*_F_ does not apply. The dots represent the average of 20 repetitions of the randomized cross-validation, and the shaded area indicates the standard deviation. The pronounced dots relate to the representative accuracy, which is defined in the text. Feature selection is abbreviated by “FS” in the legend. The quantization margin is abbreviated by “QM”.

Figure 5 gives a qualitative overview of the performance evaluation for S1–S6. At this stage, it is already evident that whitening dramatically improves decoding performance, regardless of the feature reduction technique. For CSP and feature selection, a gradual improvement can be observed in most datasets as *N*_F_ increases. This is plausible as new information is added to the system. It is remarkable that this characteristic is different for PCA-based feature reduction: more features only slightly improve performance, if there is any improvement at all. For many datasets, the best performance is already achieved for very few PCA components and degrades as more are added to the system. The representative accuracy is indicated by the large dots. Note that the concept of representative accuracy does not apply to rLDA, since it directly operates on the vectorized feature space.

Table 4 lists the representative mean accuracies, standard deviations, and respective number of features versus methods and datasets. For better reading, we ordered the presentation by protocol and electrode grid density. Below, we summarize the most important findings. For brevity, we refer to TVLDA with PCA-based feature reduction and whitening just as TVLDA.

**Table 4:** Performance overview of all assessed methods on all datasets.

			**No Whitening**				**Whitening**								
**Dataset**			**LDA**				**rLDA^**c**^**	**LDA**						**TVLDA**	
			**CSP**		**Channel Sel**.			**CSP**		**Channel Sel**.		**PCA**^****d****^		**PCA**^****d****^	
**Protocol^**a**^**	**Grids^**b**^**	**ID**	**Acc. (%)**	***N*_F_**	**Acc. (%)**	***N*_F_**	**Acc. (%)**	**Acc. (%)**	***N*_F_**	**Acc. (%)**	***N*_F_**	**Acc. (%)**	***N*_F_**	**Acc. (%)**	***N*_F_**	
RPS	Macro	S1	63.8 ± 3.0	2	52.2 ± 3.1	4	68.8 ± 2.5	73.8 ± 2.0	1	69.1 ± 0.9	1	65.4 ± 1.5	1	**82.4** ± **2.0**	**1**	
RPS	Macro	S6	53.6 ± 2.2	3	57.3 ± 1.5	9	79.9 ± 0.8	76.5 ± 1.4	8	77.6 ± 1.0	8	79.2 ± 0.8	1	**91.3** ± **0.9**	**2**	
Average			58.7 ± 2.6	2.5	54.7 ± 2.4	6.5	74.4 ± 1.9	75.2 ± 1.7	4.5	73.4 ± 0.9	4.5	72.3 ± 1.2	1.0	**86.9** ± **1.6**	**1.5**	
RPS	Micro	S2	75.9 ± 1.7	4	77.3 ± 2.2	8	89.2 ± 1.0	91.7 ± 1.2	3	82.0 ± 2.4	11	91.6 ± 0.8	1	**99.0** ± **0.3**	**1**	
RPS	Micro	S4	77.4 ± 2.3	6	79.8 ± 1.2	4	67.8 ± 1.5	90.7 ± 1.0	11	90.3 ± 1.0	13	92.5 ± 0.7	2	**98.9** ± **0.2**	**1**	
RPS	Micro	S5	87.4 ± 1.8	5	89.0 ± 1.1	7	96.3 ± 0.4	95.0 ± 0.6	4	95.7 ± 0.6	5	**98.4** ± **0.3**	**1**	**99.0** ± **0.4**	**1**	
Average			80.2 ± 2.0	5.0	82.0 ± 1.6	6.3	84.4 ± 1.1	92.5 ± 1.0	6.0	89.4 ± 1.5	9.7	94.2 ± 0.7	1.3	**99.0** ± **0.3**	**1.0**	
FTPU	Macro	S7	54.9 ± 3.6	4	60.2 ± 3.0	7	79.3 ± 1.7	65.3 ± 3.5	2	65.6 ± 2.3	4	76.0 ± 2.1	1	**89.4** ± **1.3**	**1**	
FTPU	Macro	S8	56.6 ± 1.7	1	63.5 ± 2.3	4	71.8 ± 1.9	75.6 ± 2.4	2	**79.4** ± **1.6**	**5**	69.1 ± 2.2	1	**82.8** ± **1.2**	**1**	
FTPU	Macro	S9	53.5 ± 3.4	1	70.8 ± 3.6	7	**83.3** ± **1.1**	72.9 ± 2.1	2	75.4 ± 2.1	3	78.6 ± 2.3	1	**85.7** ± **1.2**	**1**	
FTPU	Macro	S10	55.8 ± 3.2	4	62.8 ± 2.1	2	60.8 ± 1.6	57.7 ± 1.9	1	**73.9** ± **1.8**	**2**	71.0 ± 1.9	1	**77.3** ± **2.0**	**1**	⋆
FTPU	Macro	S11	27.0 ± 2.9	1	38.0 ± 2.5	3	50.4 ± 1.6	39.3 ± 2.1	1	50.8 ± 2.7	5	50.4 ± 1.9	1	**64.5** ± **3.2**	**1**	
FTPU	Macro	S12	40.0 ± 0.0	1	53.3 ± 0.0	1	70.0 ± 0.0	60.0 ± 0.0	1	63.3 ± 0.0	1	**80.0** ± **0.0**	**1**	**90.0** ± **0.0**	**1**	⋆
FTPU	Macro	S13	49.9 ± 2.7	3	57.4 ± 2.0	1	74.6 ± 1.8	66.0 ± 1.5	1	72.4 ± 1.8	3	68.1 ± 2.8	1	**80.1** ± **1.7**	**2**	
FTPU	Macro	S14	55.7 ± 0.0	2	67.1 ± 0.0	2	71.4 ± 0.0	60.0 ± 0.0	3	64.3 ± 0.0	5	**78.6** ± **0.0**	**1**	**81.4** ± **0.0**	**1**	⋆
FTPU	Macro	S15	53.5 ± 1.7	1	58.9 ± 2.3	2	58.9 ± 2.0	68.7 ± 1.4	1	68.9 ± 1.8	2	70.9 ± 1.4	1	**77.5** ± **1.7**	**1**	⋆
Average			49.6 ± 2.8	2.0	59.1 ± 2.6	3.2	68.9 ± 1.7	62.8 ± 2.2	1.6	68.2 ± 2.1	3.3	71.4 ± 2.1	1.0	**81.0** ± **1.9**	**1.1**	
FTPU	Micro	S2	77.8 ± 1.5	5	80.9 ± 1.4	4	80.2 ± 1.3	79.8 ± 1.7	7	85.3 ± 1.2	7	87.5 ± 1.1	1	**97.2** ± **0.6**	**1**	
FTPU	Micro	S3	**93.3** ± **1.8**	**2**	83.8 ± 1.1	3	85.8 ± 1.2	**95.3** ± **0.6**	**2**	**93.1** ± **0.7**	**3**	**91.0** ± **1.4**	**2**	**93.8** ± **1.4**	**1**	
FTPD	Micro	S4	67.7 ± 2.4	12	65.3 ± 1.3	8	50.5 ± 0.9	85.3 ± 0.9	5	85.0 ± 0.7	10	89.3 ± 0.5	1	**97.9** ± **0.3**	**1**	
FTPU	Micro	S4	71.0 ± 1.1	7	75.0 ± 0.9	5	51.7 ± 1.0	89.0 ± 0.7	4	88.8 ± 0.6	9	89.2 ± 0.6	1	**96.6** ± **0.3**	**2**	
FTPU	Micro	S5	58.1 ± 1.5	12	55.4 ± 1.0	9	68.2 ± 0.9	64.3 ± 1.1	8	65.2 ± 0.9	12	70.6 ± 0.8	1	**85.3** ± **0.8**	**2**	
Average			73.6 ± 1.7	7.6	72.1 ± 1.2	5.8	67.3 ± 1.1	82.7 ± 1.0	5.2	83.5 ± 0.9	8.2	85.5 ± 0.9	1.2	**94.2** ± **0.8**	**1.4**	

*The table is organized in four blocks, such that the rock-paper-scissor experiments with standard and high-density electrode grids are clustered in the first and second block, respectively. Likewise, the finger-tapping experiments with standard and high-density electrode grids are presented in the third and fourth block, respectively. Accuracies and corresponding number of features N_F_ are representative values, as described in the text. The percentages are given as means ± standard deviation over 20 cross-validation repetitions. If the standard deviation is missing, it was zero due to the low number of trials. Emphasized values are considered the best for each dataset, which lie within the quantization margin of the best result (cf. section 2.9.3)*.

a *RPS, rock-paper-scissors; FTPD, finger tapping, palm down; FTPU, finger tapping, palm up*.

b *Macro, standard ECoG grid; Micro, high-density ECoG grid*.

c *rLDA operates on vectorized features, so N_F_ is not applicable (cf. section 2.6.2)*.

d *For rows marked with an asterisk (⋆), a regularization of 25% was used for PCA (cf. section 2.7)*.

### 3.1. Relative Performance

As summarized in Table 4, the accuracies increase systematically from the standard methods to TVLDA. Whitening already has a dramatic impact on the performance. For CSP and LDA, the improvement peaks at +22.9% (S6, RPS) with +12.3% on average. A similar trend can be observed for feature selection and LDA, where we improved by up to +20.3% (S6, RPS) and +10.4% on average.

For whitened data, rLDA performed worst on average with 71.5%. LDA with CSP and feature selection was slightly better with 74.0 and 76.1% on average. LDA with PCA was the best on average with 78.8%. At the same time, PCA turned out to be the most efficient feature reduction technique by far, needing only 1.1 components on average instead of 3.5 (CSP), 5.7 (feature selection), or the whole vectorized feature space (rLDA).

Overall, the best results were seen for whitened data and TVLDA, where TVLDA outperformed rLDA by +16.4%, LDA and CSP by +13.9%, and LDA and feature selection by +11.8% on average. Investigating the impact of using TVLDA instead of LDA for PCA-based feature reduction and whitened data, we obtained an improvement of +9.1% on average. To assess the robustness of each evaluated method, we computed the standard deviation of the accuracies over 20 repetitions of the randomized cross-validation. For the non-whitened data and LDA, we obtained an average standard deviation of ±2.2 and ±2.0% for CSP and feature selection, respectively. Whitening decreased these values to ±1.6% and ±1.5%, respectively, whereas rLDA showed an overall standard deviation of ±1.4%. TVLDA slightly diminished the overall standard deviation further to ±1.3%.

### 3.2. Absolute Performance

TVLDA performed best not only on average, but for every single dataset (within the quantization margin relative to the overall maximum). Only one or two PCA features (1.1 on average) are needed to achieve top performance. For the subsequent discussion, we thus refer to the results yielded by TVLDA.

Combining the results of the rock-paper-scissors experiment for the subjects with standard ECoG grids implanted, an overall accuracy of 86.9% was achieved. In contrast, the accuracy increases tremendously for subjects with high-density grids implanted, who scored 99.0% on average.

S7–S15, all with standard electrode grids implanted, scored 81.0% on average in the finger-tapping experiments. In general, all these data comprised fewer trials; for S12, even only 6 trials were available. The entries marked with an asterisk in Table 4 were thus obtained with a regularized PCA to avoid overfitting (cf. section 2.7).

For the patients with high-density grids implanted, the classification accuracy in the finger-tapping experiment was 94.2% over all subjects. Accounting only for the subjects with substantial sensorimotor coverage (thus excluding S5), the overall score increased to 96.4%.

## 4. Discussion

### 4.1. Classification Performance

Spectral whitening during the preprocessing stage has a tremendous impact on decoding performance. On average, the accuracy rises by +12.3% for CSP and LDA and by +10.4% for feature selection and LDA. Figure 3 illustrates the reason for this huge leap: whitening balances the information with respect to frequency and therefore substantially increases the signal-to-noise ratio. Employing multi-band features in the high-gamma band (Shenoy et al., 2007; Kubánek et al., 2009; Kapeller et al., 2014; Pan et al., 2018) may have a similar positive effect on the classification performance as whitening, but this comes at the cost of an expanded feature space.

When ECoG signals are offline analyzed in the time-frequency domain, spectral whitening is well established (Miller et al., 2009). Yanagisawa et al. (2011) and Pistohl et al. (2012) directly extracted bandpower features for classification from a time-frequency signal representation (such as short-time Fourier or wavelet transforms). However, this approach is computationally demanding and may not meet real-time constraints. We therefore strongly promote the proposed time-domain whitening filter to save valuable resources.

The evidence that TVLDA outperforms LDA on high-gamma features is overwhelming: for every single dataset, TVLDA delivers the best results. The grand average accuracy improvement relative to standard methods is +16.4% (vs. rLDA), +13.9% (vs. CSP and LDA) and +11.8% (vs. feature selection and LDA). These results were obtained with mostly one (sometimes two) internal PCA components for TVLDA, whereas CSP and feature selection require 3.5 and 5.7 components, respectively. Performance thus not only gets better, but is also achieved at lower system complexity. The fact that only few PCA components are necessary to achieve maximum performance leads to remarkable robustness against overfitting; TVLDA with PCA delivers 10 × 10 cross-validation results with a standard deviation of ±1.3% on average. If very few trials are available, a regularization term to PCA as discussed in section 2.7 can be applied to further enhance stability.

Before putting our results into the context of state-of-the art research, we want to emphasize that it was not our focus to maximize the *absolute* performance of our system, but rather to investigate the impact of structural and methodological advances proposed in this manuscript. In other words, we did not employ multiple frequency bands or add other features to improve overall performance, unlike other studies to which we compare our results. We did not reject badly or differently executed trials from the datasets. In view of good responsiveness of a real-time BCI, we kept our trial window short (±0.75 seconds, relative to movement onset), whereas longer trials would have increased classification accuracies for some datasets most certainly.

The three-class rock-paper-scissors experiment with standard electrodes yielded an average accuracy of 86.9%. In view of the experiment settings, this compares best to 68.3% (Yanagisawa et al., 2011), 83.8% and 95.8% (Kapeller et al., 2014), 92.7% (Xie et al., 2015), and 80.0% (Li et al., 2017). Whereas the cited reference results relate to the same protocol in general, they were obtained from multi-band features and substantially longer trial durations. Xie et al. (2015) also used alternative features besides bandpower.

For the rock-paper-scissors experiment with high-density electrodes, TVLDA delivered almost perfect accuracies of 99.0% on average over three subjects. A similar experiment was recently conducted by Pan et al. (2018), who reported an accuracy of up to 90%. Bleichner et al. (2016) achieved 85.5% and Branco et al. (2017) attained 85.0% accuracy with high-density grids, but for an experiment involving four gestures.

For standard electrode grids and the finger-tapping experiment, TVLDA scored 81.0% on average over all subjects. This is well in line with state-of-the art results, such as such as 86.3% (Onaran et al., 2011), 80.3% (Kubánek et al., 2009), and 77.0% (Shenoy et al., 2007). For solid differentiation of five individual fingers, however, the spatial sampling of standard-sized grids may be too coarse. In particular, we observed considerable confusion between the ring finger and little finger, which are in fact difficult to move independently. Combining these two classes improved decoding performance to 88.1% in a four-class scenario, which seems a more usable setup in this context. Interestingly, this result with 88.1% is higher than the 86.9% we obtained for only three classes. We thus suspect that the electrode coverage of S1 was not particularly fortunate for the rock-paper-scissors experiment, or the movements were not executed consistently or pronounced enough. Based on our experimental evidence, we believe that the rock-paper-scissors experiment with proper sensorimotor coverage of standard-sized electrodes and good subject participation should yield around 90% accuracy and above with TVLDA (as in S6).

The finger-tapping experiment with high-density sensorimotor coverage resulted in 96.4% on average. This is comparable to Hotson et al. (2016), who scored a maximum of 96.5% in a single subject, but with posterior selection of the best LDA evaluation time point. With both standard and high-density electrodes implanted, Chestek et al. (2013) reported 79.3% in a similar experiment.

TVLDA with PCA has further advantages beyond high classification accuracy. The architecture needs only minor extensions compared to standard LDA. Additionally required components encompass a time-domain whitening filter for preprocessing, the summation over several LDA scores, temporal smoothing of sample means and covariances for PCA, and an SVD of the spatiotemporal TVLDA weight matrix. All of these elements are strictly deterministic and can be implemented easily. Training a system that implements TVLDA is fully automatic and done in less than a second. Moreover, all shortcomings and difficulties of CSPs and feature-selection approaches disappear, since no external feature reduction is required. TVLDA is more robust than any other assessed approach, even when only few trials are available. With only one or two PCA components, TVLDA already attains maximum performance.

### 4.2. Extensions, Limitations, and Outlook

Choosing the optimal number of principal components for TVLDA may be straightforward in the given context, where performance vs. number of features was evaluated via cross-validation. In fact, one could have chosen just the first principal component for all datasets with still very good results. TVLDA may however be applied to more complicated datasets, where more than one principal component is required. In this case, cross-validation is still an option to determine the optimal number of principal components. A more theoretical approach that efficiently estimates the true number of underlying principal components via Bayesian model selection was proposed by Minka (2001).

We already mentioned that the temporal independence of the noise as stated in (9) may be violated by inconsistent trial repetitions of the subject. In this case, the assumptions of a diagonal covariance matrix for TVLDA as in (24) is not justified any more. In fact, a good estimate of the *true* covariance matrix can be obtained from the training statistics of the LDA scores (22) with considerable effort. We tested this option, but it did not yield any improvements. On the contrary, TVLDA turned out to become less stable. We therefore resorted to the variant proposed in this manuscript, which can also be seen as a form of regularization.

As evidenced by Figure 3, our high-gamma band of choice covered several harmonics of the power-line frequency. Since power-line interference can be huge, especially for ECoG data, it must be addressed. Applying notch filters is a robust solution, although they remove the complete signal within the specified frequency band. As a consequence, we expect to have lost up to 10% of the signal power (harmonic spacing: 50/60 Hz, notch filter bandwidth: 5 Hz). In reality, it may be much less than 10% though, since the filter cut-offs are not infinitely steep. A more sophisticated interference cancelation approach that removes only unwanted signal components could have maintained a higher signal-to-noise ratio. This may have led to slightly better performance, especially for whitened data.

Our evaluation is based on retrospective analysis of offline data. However, since the signal processing pipeline is strictly causal, we are confident that the whole system can be put to the online context in a straightforward manner, yielding comparable results. Based on the experimental evidence and our experience with TVLDA, 20 trials per class for training should already be enough for reasonable online classification performance, provided that the coverage is good and high-density grids are used. Of course, more training data can often improve results.

It should be noted that TVLDA is trial-based per se, so it needs a trigger to perform classification. An interesting undertaking would be the adaptation of TVLDA for asynchronous BCIs. For training, triggered and labeled data will still be necessary (as for most supervised classifiers). During a free run, the previously trained, asynchronous TVLDA may then continuously process the incoming data stream in sliding windows. This yields one classification result at a time, including idle time periods. To reduce this large number of produced false positives, we suggest two strategies. First, the TVLDA scores themselves may be taken into account, such that only scores that exceed a minimum of certainty actually trigger a classifier output. This threshold may be determined during training. As an alternative, a baseline class could be added to the framework to explicitly account for the idle state.

In any case, TVLDA is a window-based classifier and thus requires a consistent spatiotemporal activation pattern for successful classification. Truly continuous BCI control may be difficult to implement with TVLDA.

In its design as proposed here, TVLDA requires each trial to be completed until it is classified. For real-time applications, the trial window should therefore be as short as possible. We can imagine however an adaptive TVLDA that does not necessarily accumulate the LDA scores over the whole trial. Rather, it would raise a classification output whenever the accumulated LDA scores up to the current time point exceed a certain threshold that allows a reliable decision.

TVLDA may also find usage in different application fields apart from classification. For example, as shown in (30), the TVLDA score relates to the accumulated Kullback-Leibler divergence, which can be used for statistical evaluation such as in trial-based functional brain mapping. Here, a particular task is usually compared to a resting condition. Applying TVLDA at each channel separately would then yield a robust measure how much the respective channel is involved in carrying out the task.

Another potential use case of TVLDA would be the reconstruction of task-related activation patterns. The PCA of the TVLDA weight matrix readily provides a decoupled spatial and temporal representation of the underlying cortical processes that are specifically discriminating between the two classes. A similar tool was published by Kobak et al. (2016), who proposed a demixing PCA (dPCA). Here, PCA was extended with task-related information to reduce data dimensionality and to reveal unique activation patterns specific to each task. Unlike TVLDA, dPCA was designed to simplify the analysis and visualization of multi-dimensional neural recordings in animals, but it may also be used for classification.

One fundamental assumption of TVLDA is that each trial is the exact repetition of each other trial. While this leads to a convenient signal model, it may not reflect reality. Most likely, the overall amplitude of the underlying activation curve may vary over trials due to adaptation, high-gamma attenuation, learning, or fatigue. It may be worthwile to study a potential extension of TVLDA that allows for these fluctuations or trends. This may be inspired by Williams et al. (2018), who have recently shown that tensor component analysis (TCA)—a multilinear extension of PCA—provides a powerful framework for decomposing triggered neural data into electrode factors, time factors, and *trial* factors.

## 5. Conclusions

In this work, we have outlined a novel classification method for invasive motor-control BCIs that extends LDA to account for time-variant features. We named it TVLDA, for time-variant linear discriminant analysis. At the same time, we proposed an optimized feature extraction path for high-gamma bandpower that utilizes time-domain whitening for improved performance. We assessed the performance of TVLDA by evaluating data from 15 epilepsy patients with implanted subdural grids. Based on 19 experiments involving three high-level gestures and individual finger movement, we systematically demonstrated the superiority of TVLDA over several reference methods based on LDA.

TVLDA establishes a new benchmark for invasive motor-control BCIs, especially for those with high-density electrodes implanted on sensorimotor areas. To our knowledge, 99.0% for the recognition of three high-level gestures and 96.4% for individual finger identification are the highest consistent accuracies ever reported for these kinds of experiments.

Among the strengths of TVLDA is its ability to dramatically reduce feature dimensionality through a novel projection scheme based on PCA. This leads to robust performance, even for experiments with very few trials. As a valuable consequence, TVLDA makes any preceding feature reduction stage obsolete. The implementation of TVLDA is straight forward and requires only few adaptations compared to standard LDA.

It is evident that TVLDA is not limited to motor-based classification tasks. Rather, it can be used for any experimental setup that produces spatio-temporal activation patterns for classification—potentially even in EEG or other non-brain imaging approaches, such as electrooculography (EOG), electrocardiography (ECG), electromyography (EMG), and the like. TVLDA may also find use in different ECoG applications, such trial-based functional brain mapping.

Overall, we believe that we have developed a valuable tool that will open the door for invasive brain-computer interfaces with almost perfect multi-class control in the near future. However, additional work is necessary to further validate TVLDA with different ECoG environments, as well as with EEG and other imaging methods for clinical and scientific applications.

## Data Availability

The recordings of S7–S15 analyzed for this study can be found in the *fingerflex* dataset provided by Kai Miller (https://stacks.stanford.edu/file/druid:zk881ps0522/fingerflex.zip).

## Ethics Statement

This study was carried out in accordance with the recommendations of the institutional review board of Asahikawa Medical University with written informed consent from all subjects. All subjects gave written informed consent in accordance with the Declaration of Helsinki. The protocol was approved by the institutional review board of Asahikawa Medical University.

## Author Contributions

JG developed the methods, was involved in data acquisition, performed data processing, and prepared the manuscript. AZ assisted in data processing, results evaluation, and interpretation. CK was involved in data acquisition and contributed to methods development. KK supervised the clinical study. JS provided scientific input. CG founded g.tec medical engineering GmbH and supervised the project.

### Conflict of Interest Statement

Several authors of this publication are employed at g.tec medical engineering GmbH, Austria. As part of a Ph.D. program, our motivation for conducting the study and developing the methods was entirely scientific. However, the described algorithms may be commercialized by g.tec medical engineering GmbH in the future.

## Footnotes

^1^https://stacks.stanford.edu/file/druid:zk881ps0522/fingerflex.zip
